# Effect of Multilayered Structure on the Static and Dynamic Properties of Magnetic Nanospheres

**DOI:** 10.1021/acsami.2c05715

**Published:** 2022-07-25

**Authors:** Conor McKeever, Mustafa Aziz

**Affiliations:** † Department of Physics and Astronomy, University of Exeter, Exeter EX4 4QL, United Kingdom; ‡ Department of Engineering, University of Exeter, Exeter EX4 4QF, United Kingdom; § MaxLLG, Exeter Science Park, Exeter EX5 2FN, United Kingdom

**Keywords:** ferromagnetic, interface, core−shell, resonance, reversal, multilayer, metamaterial

## Abstract

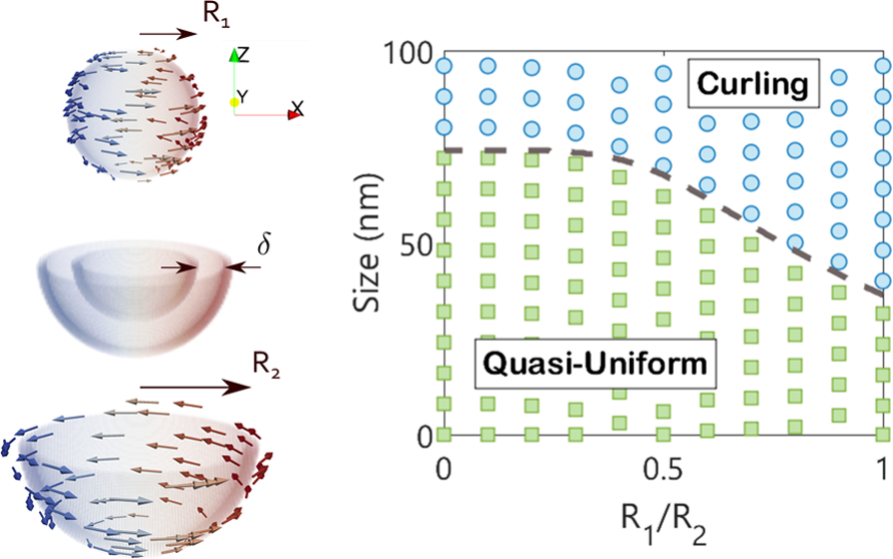

The development of flexible and lightweight electromagnetic interference (EMI)-shielding materials and microwave absorbers requires precise control and optimization of core–shell constituents within composite materials. Here, a theoretical model is proposed to predict the static and dynamic properties of multilayered core–shell particles comprised of exchange-coupled layers, as in the case of a spherical iron core coupled to an oxide shell across a spacer layer. The theory of exchange resonance in homogeneous spheres is shown to be a limiting special case of this more general theory. Nucleation of magnetization reversal occurs through either quasi-uniform or curling magnetization processes in core–shell particles, where a purely homogeneous magnetization configuration is forbidden by the multilayered morphology. The energy is minimized through mixing of modes for specific interface conditions, leading to many inhomogeneous solutions, which grow as 2^*n*^ with increasing layers, where *n* represents the number of magnetic layers. The analytical predictions are confirmed using numerical simulations.

## Introduction

Monodisperse core–shell particles possessing exchange-coupled magnetic layers have attracted considerable interest in recent decades, as the performance of microwave absorbers,^1−4^ novel spintronic devices,^5,6^ and coercivity enhancement for permanent magnets^7,8^ can be tailored based on interfacial layer coupling. Core–shell particles inherit the traits of both the core and surrounding shell, which results in enhanced surface properties that are not present when only a single material is utilized.^9−13^ By precisely controlling the layer thickness and interlayer coupling,^14−16^ it is possible to enhance the properties of core–shell particles for both existing and prospective applications. Hence, an in-depth understanding of the behavior of core–shell constituents within composite materials is key to achieving technological applications. Magnetic particles have attracted broad attention for a vast number of applications such as impedance matching and antenna miniaturization^17−19^ in addition to targeted destruction of cancer cells.^20−22^ In the latter case, it is crucial to achieve repeatability of the magnetization reversal mechanism as this determines the heating performance of the core–shell system during the hysteresis cycle.^23−26^ In the gigahertz frequency regime, exchange modes have drawn significant interest for the development of efficient microwave absorbers.^27−31^ Exchange modes are known to occur at frequencies above the uniform ferromagnetic resonance and provide a potential avenue to achieve impedance matching between magnetic/dielectric layers and to broaden electromagnetic wave absorption at high frequencies.^32,33^

However, precise control and prediction of the static and dynamic magnetic properties of multilayered particles remain a challenge due to their dependence on compositional structure, surface coatings, and interlayer coupling. Although most devices exploiting multilayered effects rely on thin films,^34,35^ increasing attention has been directed toward three-dimensional magnetic geometries, such as spherical particles,^36^ helices,^37,38^ torus,^39−41^ and the Möbius ring,^42^ as they provide new topological mechanisms for controlling magnetic properties at the nanoscale.^43,44^ Recent analytical studies of spherical shells have focused on curvature-induced magnetization textures and skyrmions,^45^ phase transitions,^46^ and magnetic resonance,^47−49^ where the shell is usually placed in contact with a vacuum at the outer/inner surfaces. However, for practical applications to be realized, it is also important to consider the static and dynamic behavior of magnetic shells when they are directly coupled to neighboring materials across a nonmagnetic interface.

In this letter, the theoretical results for nucleation and exchange resonance in homogeneous spheres are generalized to an arbitrary number of exchange-coupled magnetic layers separated by nonmagnetic interfaces. It is shown that homogeneous reversal is generally forbidden in spherical core–shell particles. The critical sizes for transitions from curling to quasi-uniform behavior are calculated, and the number of mixed nucleation modes is shown to scale as 2^*n*^, where *n* is the number of magnetic layers. The results can be computed quickly for an arbitrary number of exchange-coupled magnetic layers of finite thickness, in contrast to numerical investigations, which suffer rapid growth of adjustable input parameters with increasing layers, leading to prohibitively expensive computation time. Moreover, it is possible to study interface and surface coating effects analytically without relying on the concept of phenomenological surface anisotropy. Finally, the analytical predictions are confirmed using numerical simulations.

## Methodology

### Analytical Model

Consider a spherical particle comprised of ferro- or ferrimagnetic material and let the external field *H* be applied along the *z* axis, which is also assumed to be an easy axis for magnetocrystalline anisotropy energy and can be either cubic or uniaxial. For this system, the nucleation of magnetization reversal is described by the linearized differential equation^50^
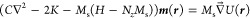
1where ***m***(***r***) is a small transverse variation of the magnetization, *M*_s_ is the saturation magnetization, *C* and *K* are the local exchange and anisotropy constants, *H* is the applied field, and *N*_*z*_ is the demagnetizing factor along the direction of saturation. *U*(***r***) represents the scalar potential created by the transverse magnetization distribution ***m***(***r***) and is described by the magnetostatic Poisson equation
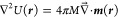
2

Two eigenmodes are of interest in the present context of core–shell particles, namely, curling and a quasi-coherent rotation mode, where coherent (Stoner–Wohlfarth) rotation is found to be forbidden. For reversal by the curling mode, the transverse demagnetizing field can be written in the closed form as ∇⃗*U*(***r***) = *NM*_s_***m***(***r***), and the system of equations is readily solved to give *H*_d_ = −4π*M*_s_/3. Although the demagnetizing field for a quasi-uniform magnetization state does not simplify in the same way, we can nevertheless treat it as a perturbation of uniform magnetization and obtain an approximate solution^51^
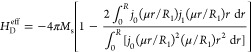
3where μ is the unperturbed eigenvalue corresponding to eqs 4.1–4.3 for the case of quasi-uniform rotation and ***m***(***r***) ∝ *j*_0_(μ*r*/*R*_1_). In the limit of a purely homogeneous configuration ***m***(***r***) → const(μ → 0), the perturbation reduces to the transverse demagnetizing factor of an ideally saturated sphere *H*_D_^eff^ = −4π*M*_s_/3. The perturbative correction to curling nucleation ***m***(***r***) ∝ *j*_1_(μ*r*/*R*_1_) vanishes due to the flux closure.

**Figure 1:**
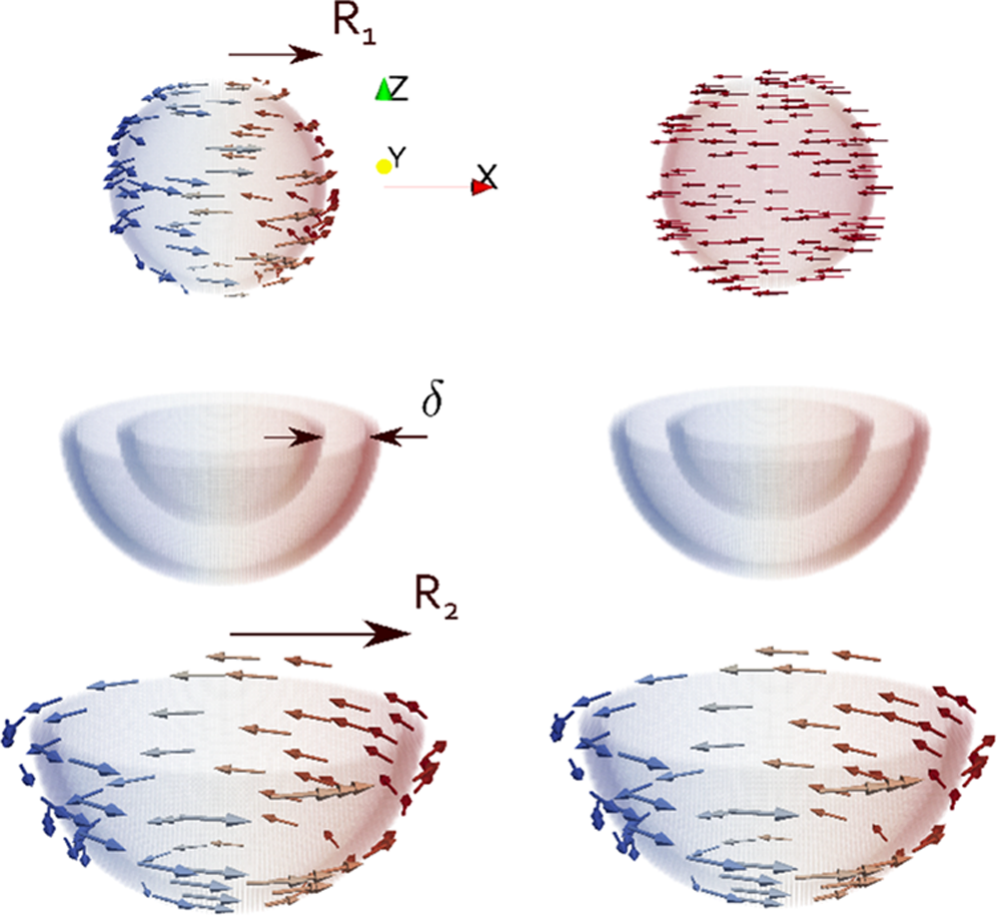
Simplified schematic of curling and quasi-uniform nucleation mechanisms in a magnetic core of radius *R*_1_ and magnetic shell of outer radius *R*_2_, separated by a thin nonmagnetic layer of thickness δ.

Subject to these constraints, it can be verified by substitution that the most general solution that satisfies the differential eqs 1 and 2 in spherical coordinates and is regular at *r* = 0 is^52^
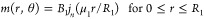
4.1
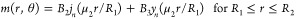
4.2
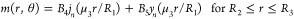
4.3where *m*(*r*,θ) represents the azimuthal component of the magnetization, μ are the eigenvalues, *R*_1_ is the radius of the core and *R*_2_ is the radius of an inner magnetic shell, each shown in Figure 1, and *R*_3_ is the outer radius of an additional outer magnetic shell. *j* and *y* are spherical Bessel functions of the first and second kind, respectively, and *B*_*i*_ are arbitrary integration constants. The physical form of the solution depends on the specific material, geometric, and interfacial parameters. When the eigenvalues have complex solutions, the spherical Bessel functions are replaced with their modified forms *I*_*n*_ and *K*_*n*_, which represent localized decaying modes. Substituting the solutions (eqs 4.1–4.3) into the differential eqs 1 and 2 leads to three equations for the nucleation field
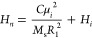
5where *i* = 1, 2, 3 represent the effective field of the spherical core, an inner shell, and an outer shell, respectively. To calculate the nucleation field *H*_*n*_ of a given mode from eq 5, it is necessary to first determine the eigenvalues μ_*i*_ by imposing the boundary conditions and continuity of the magnetization at the material interfaces.

Continuity of the magnetization at the core–shell *r* = *R*_1_ interface is assumed, which leads to the following equation
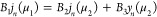
6

The generalized Barnaś–Mills boundary conditions are adopted here to describe the exchange coupling of a ferromagnetic core to an outer magnetic shell across a nonmagnetic interface of finite thickness^53,54^
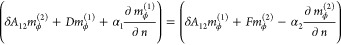
7

where *D* = −[(*A*_12_ – β_12_)ζ – β_1_]δ, *F* = −[(*A*_12_ – β_12_)/ζ + β_2_]δ, and ζ = *M*_2_/*M*_1_. The interlayer exchange coupling can have different origins, including Ruderman–Kittel–Kasuya–Yosida (RKKY) interactions. The exchange and magnetocrystalline anisotropy energy densities are denoted by α = *C*/2*M*_s_^2^ and β = *K*/*M*_s_^2^, respectively. *A*_12_ = ξ*A*_int,s_/(*M*_int_^2^ δ) is a parameter of uniform exchange interaction, where *A*_int,s_ denotes the effective surface exchange constant of the interface *A*_int,s_ = *A*_int_/δ with *A*_int_ being the exchange constant of the interface region and δ the interface thickness. *M*_int_ is the effective saturation magnetization of the interface and ξ is a proportionality constant, which can be derived in principle from ab initio calculations. The parameter β_12_ = *K*_12_/*M*_int,s_^2^ is an anisotropy parameter, where *K*_12_ is the uniaxial magnetic anisotropy at the interface. Although the particle has been physically divided into multiple layers, the continuity of the effective magnetization across the exchange-coupled interface preserves the overall spherical shape of the particle from a mathematical point of view. The physical interpretation of *D* and *F* is that of effective material properties obtained by averaging over the finite thickness δ of the interface. If the inner shell is exchange-coupled directly to an additional outer shell at the interface *r* = *R*_2_, then continuity of the magnetization across this interface leads to another equation
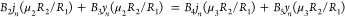
8

The derivative *C*_2_(∂ *m*_2_)/∂ *n* = *C*_3_(∂ *m*_3_)/∂ *n*^55^ of the magnetization at the coupled inner–outer shell interface must also be continuous at *r* = *R*_2_, hence
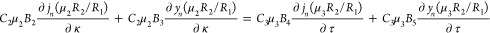
9where κ = μ_2_*R*_2_/*R*_1_ and τ = μ_3_*R*_2_/*R*_1_. Finally, at the outer surface of the outer shell, the usual boundary condition for free surface spins is chosen ∂ *m*/∂ *n* = 0, yielding
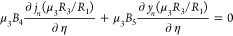
10where η = μ_3_*R*_3_/*R*_1_. Nontrivial solutions satisfying these eqs 6–10 exist if the determinant of the coefficients *B*_1–5_ vanishes. This determinant can be solved with the three expressions (eq 5) to compute the magnetization reversal mode for a given set of parameters. Matrix equations are presented in the Supporting information file for magnetic cores with one and two coupled shells. By repeating the above procedure for additional boundary conditions and eigenvalues, it is straightforward to generalize the solution to an arbitrary number of shells with varying material parameters. For the linear nucleation problem, only *n* = 0, 1 are of physical interest and nucleation occurs through the smallest mode only. After nucleation, large-angle deviation of *M* from saturation, beginning at the nucleation field, is a typical bifurcation phenomenon in nonlinear functional analysis.^56,57^ For the exchange resonance problem, every mode can be excited when subjected to suitable excitation conditions, and mode mixing occurs when the equations are solved for different values of *n* in separate layers.

For the dynamic case, the linearized differential equations for a spherical particle in the exchange approximation are given by the equations^27^
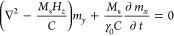
11.1
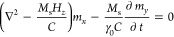
11.2

The most general solution of this system of equations is^48,58^
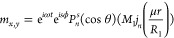
12
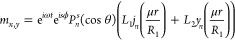
13in the spherical core and in the shell regions, where *M* and *L* are real constants, *s* and *n* are integers, and *P*_*n*_^*s*^ are the Legendre functions. Substituting these solutions into the differential equations and repeating the previous procedure to define the boundary conditions and continuity of the magnetization at material interfaces lead to the same matrix expression for the eigenvalue equation, with three equations for the exchange resonance frequency
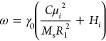
14where *i* = 1, 2, 3 corresponds to the spherical core, an inner shell, and an outer shell, respectively. The analytical expression for exchange resonance has been shown to accurately predict the resonance absorption peaks of conducting magnetic pillars subject to plane wave excitation, provided that the length of the pillar is comparable to the magnetic skin depth (approximately 50 nm for Co^33^).

### Computational Details

To investigate finite magnetic objects, coupled electromagnetic–micromagnetic simulations were performed.^33,59^ The electromagnetic fields are computed from Maxwell’s curl equations
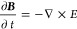
15
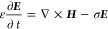
16where ***H*** is the magnetic field, ***E*** is the electric field, ε is the permittivity, σ is the electrical conductivity, and *t* is the time. The magnetic flux density ***B*** is coupled to the magnetization ***M*** through the constitutive relation ***B*** = μ_0_(***M*** + ***H***), where μ_0_ is the permeability of free space. The magnetization is evaluated from the solution of the Landau–Lifshitz–Gilbert equation (LLG)
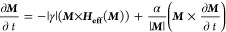
17where γ = 1.75882 × 10^11^μ_0_ (m·Hz·A^–1^) is the gyromagnetic ratio, α is the phenomenological Gilbert damping coefficient, and |***M***| = *M*_s_ is the saturation magnetization. To achieve rapid convergence during static calculations, the conductivity is artificially increased to accelerate the damping of the electric fields. The effective field ***H***_**eff**_ in eq 17 is given by
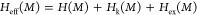
18

where ***H*** is the Maxwell field evaluated from the solution of eq 15, the anisotropy field ***H***_**κ**_ = −*gM*_z_*Z* describes the uniaxial anisotropy along the *z* axis, where *g* = 2*K*_1_/μ_0_*M*_s_^2^ and *K*_1_ is the anisotropy constant. ***H***_**ex**_ in eq 18 is the nearest neighbor exchange field contribution , where ∇^2^ is the Laplacian operator. The parameters used in the simulations are taken from Table S1.

## Results and Discussion

Recent work has shown that exchange-coupled core–shell particles exhibit mixed reversal processes with uniform-like or vortex-like magnetic configurations emerging depending on the specific material, geometric, and interfacial properties of the particle.^60^ Vortex configurations, which are typically found in larger particles, have shown enhanced specific absorption rate (SAR) values as they reduce stray fields and diminish the formation of aggregates. Stabilization of such configurations down to smaller sizes is therefore desirable from the point of view of minimizing agglomeration and improving heating efficiency.

**Figure 2:**
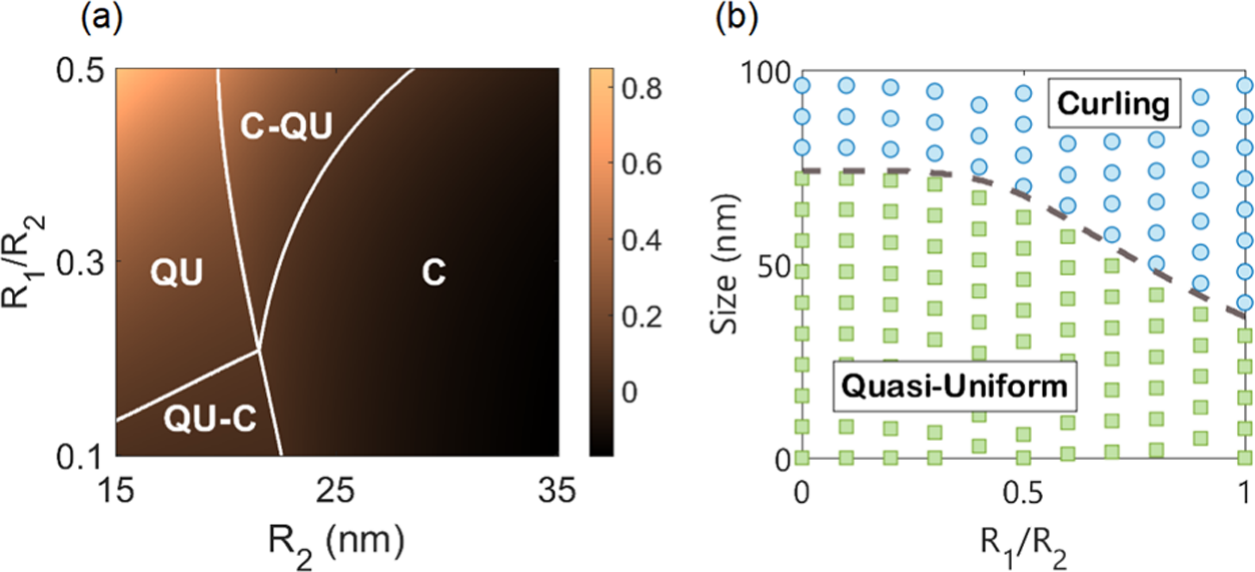
(a) Nucleation field (*T*) for a spherical magnetic core exchanged-coupled to an outer magnetic shell across a nonmagnetic layer. Modes are curling (C), quasi-uniform (QU), C (core)–QU (shell), and QU (core)–C (shell). (b) Numerical comparison between theory and a core–shell particle of the same parameters. Parameters for (a) and (b) can be found in Tables S1 and S2, respectively.

The critical size for transition between nucleation processes is shown in Figure 2a for four different modes, represented by the white lines. The largest particles permit only curling reversal, whereas the smallest permit only quasi-uniform behavior. In the intermediate size range, there is a mixed combination of curling and quasi-uniform reversal in the core and shell. When designing core–shell systems to produce repeatable reversal processes, the intersection between all four modes in Figure 2a should be avoided as small variations in particle geometry, surface roughness, or material properties will lead to significantly different magnetization processes. For other applications, a wide range of tunability based on geometric flexibility may be desirable. In Figure 2a, when the Co_80_Ni_20_ core is coupled to a Fe_14_Co_43_Ni_43_ shell on the surface, the curling mode in the core can be stabilized down to significantly smaller sizes, as shown by the growing region of “C–QU” with decreasing *R*_1_/*R*_2_. It can be confirmed by substitution that the multilayered structure prevents the formation of a purely uniform magnetization configuration because the constant function (*m*(*r*,θ) = const) does not simultaneously satisfy (eqs 1 to 4.1–4.3) and the boundary conditions (eqs 6–10) for the cases considered here, except in the limit of a homogeneous sphere where the Stoner–Wohlfarth relation is reproduced. A comparison between the analytical model and numerical simulations is shown in Figure 2b, where the quasi-uniform magnetic state becomes stable at larger particle sizes as the saturation magnetization *M*_s_ decreases throughout the particle with *R*_1_/*R*_2_ → 0. The transition between coherent rotation and curling for a homogeneous sphere is given by the equation , where *q*_2_ ≈ 2.0816.^52^ Substituting the values used in Figure 2b for the two homogeneous limits of *R*_1_/*R*_2_ = 0 and *R*_1_/*R*_2_ = 1 gives transition sizes of 2*R*_c_ = 71.918 nm and 2*R*_c_ = 35.959 nm, respectively, in exact agreement with the theoretical values shown in Figure 2b.

**Figure 3:**
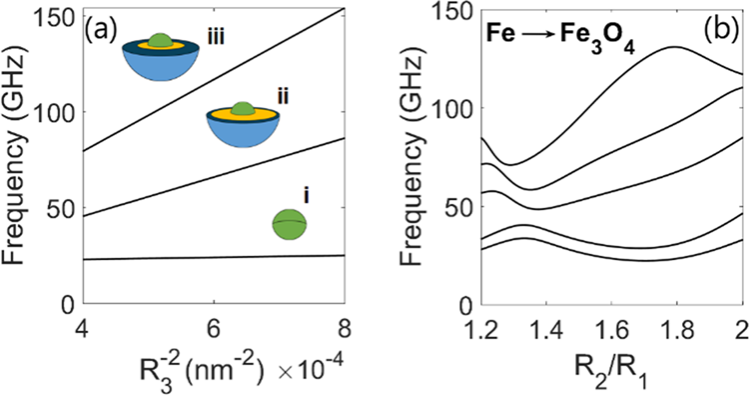
(a) Size dependence of core–shell particles with different material compositions: (i) solid cobalt sphere and a multilayered particle with cobalt (core), iron (inner shell), and magnetite (outer shell) with parameters (ii) *R*_1_ = 0.3*R*_3_, *R*_2_ = 0.8*R*_3_, and (iii) *R*_1_ = 0.3*R*_3_, *R*_2_ = 0.4*R*_3_. (b) Frequency dependence of lowest nonmixed exchange modes for an iron core coupled to an outer oxide shell. The thickness of the oxide layer on the surface increases with the increasing ratio of *R*_2_/*R*_1_. Parameters can be found in Table S3.

The size dependence of multilayered nanoparticles produced for different layer thicknesses is shown in Figure 3a using the analytical model. The presence of an oxide shell has a large impact on the resonance frequency, pushing the mode toward higher frequencies due to the decreased saturation magnetization and interlayer pinning. For small or negligible surface and interfacial layer pinning, the characteristic 1/*R*_3_^2^ power law is preserved for a multilayered particle provided that the ratio of the material is kept constant with decreasing size. Here, the dependence on the radius is determined not only by the usual frequency equation where the core radius *R*_1_ is present in the denominator (eq 12) but also by the determinant of the matrix equation where the outer radii *R*_2_ and *R*_3_ of each shell also appear. The dependence of the frequency on the outer shell radius *R*_3_ is introduced only as a function inside the matrix equation, and hence the combination of spherical Bessel functions in the determinant produces the correct inverse square law, even though it is not directly imposed at any point. The effect of a thin oxide layer that gradually grows in thickness is shown in Figure 3b using the analytical model. The resonance shows a complex dependence on the thickness of the oxide layer, due to the competing surface and material contributions, including anisotropy, exchange, and saturation magnetization. In the limit of a homogeneous sphere, the dynamic equations reduce to the exchange resonance theory developed by Aharoni.^27^

## Conclusions

In this article, a simultaneous study of magnetization reversal and ferromagnetic resonance in multilayered spherical particles was proposed, revealing the underlying static and dynamic mechanisms. The critical sizes for transitions between curling and quasi-uniform magnetic behavior, the dependence on interfacial coupling, material properties, and geometry, and the growth of permissible modes with increasing layers were obtained. We therefore establish the possibility to extract interfacial characteristics of core–shell materials by fitting to experimental data using, for example, static exchange-spring measurements or FMR studies. This work may also prove useful as a guide for numerical computations, which can mistake saddle points for global minimum during magnetization reversal, or fail to minimize the energy sufficiently close to infinitesimal instabilities, resulting in serious errors in main or minor hysteresis jumps depending on the system. The theory was confirmed numerically and reproduces well-known limits of homogeneous spheres, and hence it can be used to support the precise design and optimization of flexible and lightweight electromagnetic interference (EMI)-shielding materials and microwave absorbers based on core–shell constituents.

## Supporting Information Available

Additional analytical details and material parameters, including exact eigenvalue equations (PDF)
