# Supplementary material for: Effect of Multilayered Structure on the Static and Dynamic Properties of Magnetic Nanospheres
Source: ACS Appl Mater Interfaces. 2022 Jul 25;14(30):35177–83. doi: 10.1021/acsami.2c05715 (PMC9354015; doi:10.1021/acsami.2c05715)
Supplement: Supplementary file 1 — am2c05715_si_001.pdf [file am2c05715_si_001.pdf]

Supporting Information

Effect of Multilayered Structure on the Static and Dynamic Properties of Magnetic Nanospheres

Conor McKeever [1,3]* and Mustafa Aziz [2,3]

[1]*Department of Physics and Astronomy, University of Exeter, Exeter, EX4 4QL, United Kingdom*

[2]*Department of Engineering, University of Exeter, Exeter EX4 4QF, United Kingdom*

[3]*MaxLLG, Exeter Science Park, Exeter EX5 2FN, United Kingdom*

Keywords: *Ferromagnetic, Interface, Core-Shell, Resonance, Reversal, Multilayer, Metamaterial*

Email@ c.mckeever2@exeter.ac.uk

$$\begin{vmatrix} j_n(\mu_1) & j_n(\mu_2) & y_n(\mu_2) \\ C_1\mu_1 j'_n(\mu_1) & C_2\mu_2 j'_n(\mu_2) & C_2\mu_2 y'_n(\mu_2) \\ 0 & j'_n(\mu_2 R_2/R_1) & y'_n(\mu_2 R_2/R_1) \end{vmatrix} = 0 \quad (1)$$

$$\mu_1 = \sqrt{\frac{M_{s1}R_1^2}{\gamma_0 C_1}(\omega_n - H_{z1})} \quad (2)$$

$$\mu_2 = \sqrt{\frac{M_{s2}R_1^2}{\gamma_0 C_2}(\omega_n - H_{z2})} \quad (3)$$

$$\begin{vmatrix} j_n(\mu_1) & j_n(\mu_2) & y_n(\mu_2) \\ -\delta A_{12} j_n(\mu_1) + D j_n(\mu_1) + & -\delta A_{12} j_n(\mu_2) + C j_n(\mu_2) - & -\delta A_{12} y_n(\mu_2) + C y_n(\mu_2) - \\ \alpha_1 \frac{\mu_1}{R_1} j'_n(\mu_1) & \alpha_2 \frac{\mu_2}{R_1} j'_n(\mu_2) & \alpha_2 \frac{\mu_2}{R_1} y'_n(\mu_2) \\ 0 & j'_n(\mu_2 R_2/R_1) & y'_n(\mu_2 R_2/R_1) \end{vmatrix} = 0 \quad (4)$$

$$\mu_1 = \sqrt{\frac{M_{s1}R_1^2}{\gamma_0 C_1}(\omega_n - H_{z1})} \quad (5)$$

$$\mu_2 = \sqrt{\frac{M_{s2}R_1^2}{\gamma_0 C_2}(\omega_n - H_{z2})} \quad (6)$$

$$\begin{vmatrix} j_n(\mu_1) & j_n(\mu_2) & y_n(\mu_2) & 0 & 0 \\ C_1\mu_1 j'_n(\mu_1) & C_2\mu_2 j'_n(\mu_2) & C_2\mu_2 y'_n(\mu_2) & 0 & 0 \\ 0 & j_n(\mu_2 R_2/R_1) & y_n(\mu_2 R_2/R_1) & j_n(\mu_3 R_2/R_1) & y_n(\mu_3 R_2/R_1) \\ 0 & C_2\mu_2 j'_n(\mu_2 R_2/R_1) & C_2\mu_2 y'_n(\mu_2 R_2/R_1) & C_3\mu_3 j'_n(\mu_3 R_2/R_1) & C_3\mu_3 y'_n(\mu_3 R_2/R_1) \\ 0 & 0 & 0 & j'_n(\mu_3 R_3/R_1) & y'_n(\mu_3 R_3/R_1) \end{vmatrix}$$

$$= 0 \quad (7)$$

$$\mu_1 = \sqrt{\frac{M_{s1}R_1^2}{\gamma_0 C_1}(\omega_n - H_{z1})} \quad (8)$$

$$\mu_2 = \sqrt{\frac{M_{s2}R_1^2}{\gamma_0 C_2}(\omega_n - H_{z2})} \quad (9)$$

$$\mu_3 = \sqrt{\frac{M_{s3}R_1^2}{\gamma_0 C_3}(\omega_n - H_{z3})} \quad (10)$$

Table of Contents Graphic

Parameters	Core	Shell
M_s	8×10^5 A/m	4×10^5 A/m
C	2×10^{-11} J/m	2×10^{-11} J/m
K_1	0 J/m^3	0 J/m^3

Table S1: Magnetic Core and Shell Exchange-Coupled at their Interface, (1)-(3); used to produce Figure 2(b). Simulations are performed using an outer radius of 25 cells for each sphere.

Table of Contents Graphic

Parameters	Core	Shell	Interface
M_s	12×10^5 A/m	8.5×10^5 A/m	-
C	4.2×10^{-11} J/m	3.8×10^{-11} J/m	-
K_1	6.4×10^4 J/m^3	1.2×10^4 J/m^3	-
ξ	-	-	-1
δ	-	-	2 nm

Table S2: Magnetic core and shell exchange-coupled across a thin non-magnetic interface of finite thickness, (4)-(6); used to produce Figure 2(a). The effective parameters of the interface were taken as averages of the magnetic properties in the core and shell.

Table of Contents Graphic

Parameters	Core	Inner Shell	Outer Shell
M_s	14×10^5 A/m	17×10^5 A/m	2.5×10^5 A/m
C	6×10^{-11} J/m	4.2×10^{-11} J/m	5.28×10^{-11} J/m
K_1	5.2×10^4 J/m^3	4.7×10^4 J/m^3	2.5×10^4 J/m^3

Table S3: Magnetic core with an inner and outer magnetic shell exchange-coupled at their interfaces, (7)-(10); used to produce Figures 3.

The unknown variables μ_i in the determinant can be eliminated using the nucleation or resonance equations, e.g. by substituting equations (2) and (3) into (1) and solving for ω. After substitution, the determinant becomes the central frequency equation which can be solved for any given value of n. Any given solution n in the core region,

is exchange-coupled to the infinite set of solutions in the shell regions, and vice versa. This leads to an expanded bandwidth which can be interpreted as a form of frequency broadening due to the multilayered structure. Adding a magnetic shell adds two rows and two columns to the eigenvalue matrix, and an additional equation which is used to determine the nucleation or resonance field.
